# Psychological symptoms and quality of life after repeated exposure to earthquake: A cohort study in Italy

**DOI:** 10.1371/journal.pone.0233172

**Published:** 2020-05-12

**Authors:** Francesco Altamore, Iolanda Grappasonni, Neelam Laxhman, Stefania Scuri, Fabio Petrelli, Giuliana Grifantini, Pamela Accaramboni, Stefan Priebe

**Affiliations:** 1 Unit for Social and Community Psychiatry (WHO Collaborating Centre for Mental Health Services Development), Queen Mary University of London, London, United Kingdom; 2 Department of Biomedical, Metabolic and Neuronal Sciences, Section of Psychiatry, Universita degli Studi di Modena e Reggio Emilia, Modena, Italy; 3 Department of Medicinal and Health Products Sciences, University of Camerino, Camerino, Italy; 4 Mental Health Department Camerino, Camerino, Italy; University of Belgrade, SERBIA

## Abstract

In 2005, a random sample of 200 people were assessed in Camerino, Italy, eight years after an earthquake. Psychological symptom levels were low and only one person had current Post-Traumatic Stress Disorder (PTSD). In 2016 a new earthquake occurred in Camerino. The study aims to assess the impact of the second exposure in the same cohort. A longitudinal study was conducted, 130 participants were re-interviewed between July and December 2017. Psychological symptoms were self-rated on the Brief Symptom Inventory (BSI) and the Global Severity Index (GSI) was analysed. Post-traumatic stress symptoms were self-rated on the Impact of Event Scale-Revised (IES-R). Subjective quality of life (SQOL) was assessed on the Manchester Short Assessment of Quality of Life (MANSA). Mean scores of GSI and IES-R were significantly higher than in 2005 (p<0.01 and p<0.001), whilst SQOL remained almost unchanged (p = 0.163). In 2017, 16.9% of the sample had reached the PTSD threshold whilst in 2005 only the 0.5% had reached it. Despite low symptom levels several years after an earthquake, people can show psychological distress after a new exposure, whilst average quality of life levels are not affected.

## Introduction

Exposure to earthquakes can lead to lasting psychological symptoms, often diagnosed as Post-Traumatic Stress Disorder (PTSD), and impaired quality of life [1–3]. Yet, most studies focus on sequelae of one event rather than repeated earthquakes as they can occur in earthquake affected regions [4,5]. A recent study in adolescents in China analysed sequelae of two earthquakes that occurred five years apart and suggested that the experience of one earthquake may increase the sensitivity to the impact of subsequent ones [6]. To our knowledge, there are no studies that investigate sequelae of repeated exposure among adults.

In the small town of Camerino in central Italy the last major earthquakes in the past two centuries occurred in 1997 and 2016. In 1997 they had a strength of 6 and in 2016 of 6.5 on the Moment Magnitude Scale [7]. Both earthquakes caused deaths in the area around Camerino. In Camerino itself, several buildings collapsed but there were no fatalities.

A 2005 survey of 200 randomly sampled people assessed the effects on mental health and quality of life eight years after the 1997 earthquake. Levels of general psychological symptoms were very low. Only one person met the criteria for current PTSD and subjective quality of life was favourable. The findings suggest that the experience of the earthquake had no substantial negative impact on psychological symptoms in the long- term [8]. This finding was unusual, particularly as studies have reported high psychological symptoms 5–10 years after traumatic exposure in the Balkans [9].

Several factors such as strong and supportive social cohesion in a rural community and a weak earthquake were considered as contributing factors to these low levels of mental distress.

The present study aims to assess how the same people who had positive long-term outcomes after the first experience of serious earthquakes would respond after exposure to another earthquake. We therefore re-assessed participants of the same cohort that was assessed in 2005 again after their experience of the 2016 earthquakes.

## Methods

### Sample and design

We conducted a longitudinal study, following up the cohort that had been identified through a random walk method and were assessed in 2005. Details and findings were described in a previous publication. We tried to contact all participants of the original study and re-assessed them between July and December 2017, i.e. between six and eleven months after the last of the new series of earthquakes. The interviews were conducted by the same research team as in 2005. Individuals were contacted by phone and interviewed face-to-face. Data was collected using paper format questionnaires that were completed by members of the research team whilst with the participant. This study was approved by the ethics committee of the Marche Region (CERM-2018 64 F99-F99).

### Measure

Socio-demographic characteristics (gender, age, school education, living situation, employment status) were recorded. Psychological symptoms were self-rated on the Brief Symptom Inventory (BSI) The BSI is a 53 item self- report scale that is rated using a 5 point Likert scale, 0 = Not at all, 4 = Extremely. We analysed the Global Severity Index (GSI), which is the mean score per item on the scale [10]. Good reliability that ranges from .71 on Psychoticism to .85 on Depression and validity ranging from .92 to .99 has been reported [10]. Post-traumatic stress symptoms were self-rated on the 22-item Impact of Event Scale-Revised (IES-R) strong reliability and validity has been reported, ranging from .89 to .94 [11]. Items are rated on a 5 point Likert scale, 0 = not at all, 5 = extremely, where scores can range from 0–88. We considered a total score of 33 as a threshold for a positive screening for PTSD [11]. Finally, subjective quality of life (SQOL) was assessed on the Manchester Short Assessment of Quality of Life (MANSA). The MANSA consists of 16 items, which is rated using a 7 point Likert scale, 1 = Couldn’t be worse, 7 = Couldn’t be better. The MANSA has good reliability with a reported Cronbach’s alpha of 0.74 and strong validity, 0.82. The mean score of the satisfaction ratings was used to reflect overall SQOL [12].

### Analysis

Socio-demographic characteristics were analysed with descriptive statistics. T-tests were used to explore differences in mean scores of the GSI, the IES-R and the MANSA between the two surveys. All analyses were conducted on SPSS version 24.

## Results

In the follow-up assessment, 130 people of the original sample of 200 were re-assessed (response rate = 65%). Eight people had died, 23 were not contactable and 39 refused to take part. Of the 130 re-assessed people, 59% were male and age ranged from 35 to 76 years; 73% were in employment, and 27% retired, unemployed or studying; 73% were married and 27% either unmarried, widower or divorced. The distribution of socio-demographic characteristics in the re-assessed 130 sample were similar to the ones in the larger sample assessed in 2005. Whereby, 58.5% were male and age of respondents ranged from 30 to 65 years; 82.5% were in employment, and 15% retired, unemployed or studying; 75.5% were married and 22.5% either unmarried, widower or divorced.

The scores on the administered scales are shown in Table 1

**Table 1:** Mean scores of general psychological symptoms, PTSD symptoms and subjective quality of life in 2005 and 2017.

Measures	2005 N = 200	2017 N = 130	t	p- value	Range of scores
Psychological Symptoms (Global Severity Index)	0.29(SD = 0.30)	0.47 (SD = 0.47)	-4.19	<0.01	0–4
PTSD symptoms (Impact of Event Scale–Revised)	0.40 (SD = 3.32)	17.81(SD = 18.27)	-13.16	<0.001	0–88
SQOL (MANSA mean score)	5.26 (SD = .60)	5.25 (SD = 0.69)	0.211	0.163	1–7

*P* values were calculated using a t-test

In 2017, mean scores of the GSI and IES-R were significantly higher than in 2005, whilst SQOL remained almost unchanged. In 2005, only one out of 200 people (0.5%) had reached the threshold for PTSD of 33 on the IES-R. In 2017, this applied to 22 out of 130 (16.9%). In a post-hoc analysis, those 22 people were found to have a poorer SQOL (MANSA = 4.64, SD = 0.67) than the 108 without PTSD (5.37, SD = 0.63, t = 4.93, p<0.001).

## Discussion

### Main findings

The study followed up a representative cohort of people living in an earthquake affected region in Italy. Whilst the interviewees had shown no raised levels of psychological distress in 2005, i.e. about eight years after experiencing serious earthquakes, their levels of psychological symptoms in general and of post-traumatic stress symptoms specifically were significantly higher in 2017, when they were re-assessed between six and eleven months after exposure to another series of similar earthquakes. The prevalence of probable current PTSD cases rose from 0.5% in 2005 to 16.9% in 2017. Yet, the overall SQOL remained similar, but was less favourable in people with PTSD.

### Strengths and limitations

This is an unusual cohort study following up a sample 12 years after the first interview. The original sample was recruited using a random walk method and may therefore be regarded as representative. The response rate of 65% in the follow-up interview may be seen as reasonably high, given the long period of time between the two interviews. Since 22 of 130 people at follow-up were screened positively for PTSD–as compared to only one out of 200 in 2005 –a possible selection bias at follow-up is unlikely to have influenced the overall finding of raised psychological distress.

The main limitation of the study is that the two interviews were done at different lengths of time since the exposure to earthquakes, as psychological symptoms may decline with the passage of time [13]. In 2005 eight years had expired since the exposure, whilst in 2017 it was less than one year. Thus, it is difficult to disentangle the impact of the shorter period of time since the earthquake from the repeated experience. Yet, despite the shorter time frame in assessing participants after the second exposure, the minimum 6 follow-up period since exposure to the second earthquake reflects the criteria for PTSD. The ICD-10 research diagnostic criteria states [14], the PTSD criteria must be met within 6 months after exposure to a stressful event. Whether the long-term response after repeated exposure is as positive as after the first exposure, could be addressed in a further follow-up in eight years time.

### Comparison with the literature

The prevalence rate of probable PTSD cases of 16.9% is similar to the PTSD prevalence of 14.3% found six months after earthquakes in Molise, another rural area in Italy struck by similar earthquakes in 2002 [15]. However, there is a large variance between the PTSD prevalence rates reported 6 months after earthquake exposure [16]. For instance, a much lower PTSD prevalence rate of 4.5% has been reported six months after an earthquake in China, whilst a study in L’Aquila, a town in a neighbouring Italian region struck in 2009 by earthquakes with about 300 fatalities, reported a prevalence rate of 56% six months after the events [17]. A systematic review and meta-analysis of studies assessing PTSD prevalence rates within nine months after earthquakes found an average rate of 28.8% [18]. Thus, the mental sequelae of the experience of the earthquakes in Camerino in 2016 is within the wide range of other findings in the literature and similar to that in Molise, yet still considerably lower than the average of other PTSD prevalence rates in the literature.

The literature suggests older age may be a risk factor for psychological distress following earthquakes [15]. Obviously, interviewees were almost 20 years older when experiencing the new earthquakes in 2016 as compared to the original ones in 1997. Yet, the age difference alone is unlikely to explain the significant increase in overall psychological distress and PTSD symptoms.

## Conclusion

The increase in symptom levels and PTSD prevalence may be due to the repeated exposure or the shorter period of time between the last earthquake and collecting data or both. In the interpretation of the very low symptom levels found in the 2005 interviews, several factors were considered as being potentially helpful in reducing the likelihood of developing symptoms. These included the supportive role of social cohesion in rural Italian communities and the fact that nobody died as a result of the earthquake in Camerino. However, it is important to note that whichever factors explained the low symptom levels in 2005, they did not protect the same people from experiencing much higher psychological distress after another earthquake series in 2016. However, perhaps these protective factors may have led to more positive long-term outcomes in 2005. The renewed exposure led to significant levels of general psychological and PTSD symptoms. Therefore, the positive long-term response after one earthquake may not be seen as a protective factor in experiencing positive outcomes after another exposure, and so, reporting low psychological distress after one earthquake does not prevent PTSD after exposure to another.

Whilst the increased psychological distress appears not to have impacted on the average SQOL, people with significant PTSD symptoms showed a lower SQOL. One can only speculate as to whether the factors that had been considered as helpful for long-term outcomes after the first earthquakes might not have been sufficient to prevent short-term distress and lower SQOL in at least some people, but have helped many others to avoid negative consequences of the experiences for the overall quality of their lives. We could also speculate that perhaps, the consistent quality of life score from 2005 through to 2017 reflects both personal and social resources that have helped the positive long-term outcome after exposure to the first earthquake, may also lead to a positive long-term outcome after the second exposure if assessed after 8 years too. We could also consider that the low level of symptoms reported in both the 2005 and 2017 samples are not due to long-term positive responses, but rather that the earthquakes were too weak to produce any long-term consequences. Thus, the low scores reported may not be a sign of coping, but rather that the psychological impact of the earthquakes was not severe enough. Perhaps, only stronger earthquakes may impact long-term outcomes as reported in the original paper. Therefore, the reported PTSD (16.9%) in the 2017 sample would perhaps have been similar even if they had not experienced an earthquake in 1997, particularly as the symptom level reported after the second exposure is still lower than that reported in other studies mentioned above. And so, the IES-R can be seen as an instrument that is sensitive to low levels of stress, which may decline over time, leaving no long-term consequences that may influence the response to new trauma.

Future research may develop a better understanding of the resilience and resources that help people sustain a good quality of life despite being distressed by the experience of natural disasters.

## Supporting information

S1 Dataset (XLSX)
